# EDITORIAL COMMENT: LAPAROSCOPIC SINGLE PORT CYSTOLITHOTOMY USING PNEUMOVESICUM

**DOI:** 10.1590/S1677-5538.IBJU.2016.0337.1

**Published:** 2016

**Authors:** David J. Hernandez

**Affiliations:** 1 Urologic Malignancies, Robotic Surgery, BPH & Urolithiasis, Tampa General Hospital, Florida, USA

Bladder stones account for ~5% of all urinary tract stones and are associated with bladder outlet obstruction, neurogenic or augmented bladders, infection or foreign bodies. Small bladder stones can be managed efficiently by transurethral methods and larger stones by open or laparoscopic approaches. However, the optimal management of multiple intermediate sized stones is controversial. Options include cystolithotomy (open or laparoscopic) and endoscopic cystolithotripsy either via a transurethral, percutaneous or combined approach using holmium:yttrium-aluminum-garnet (Ho:YAG) laser, ultrasonic or pneumatic cystolithotripsy (1, 2). When bladder stone fragmentation is necessary, I prefer the Ho:YAG laser using either a cystoscope or nephroscope as it is safe, highly effective and enables stone fixation against the bladder wall. A recent randomized, prospective study found that Ho:YAG was more effective than pneumatic cystolithotripsy for treating bladder stones smaller than 1.5 cm (3). Another randomized study comparing the three endoscopic modalities (transurethral use of cystoscope or nephroscope and percutaneous cystolithotripsy) found the transurethral route using a nephroscope to be the most efficient modality (i.e. shorter operative time) with long-term urethral stricture rate similar to transurethral cystoscope technique, but all three techniques were equally efficacious in treating bladder stones 1-4 cm (4).

Choi and Bae (5) present here a modified percutaneous cystolithotomy by the use of pneumovesicum which has been previously used for bladder cuff excision during nephroureterectomy, ureteral reimplantation for vesicoureteral reflux, and simple prostatectomy. It appears to be a safe and efficient procedure with acceptable morbidity and may be a viable alternative to the aforementioned procedures. This approach should be reserved for selected cases with stones too large for transurethral removal but small enough to be readily extracted via a laparoscopic port.

***David J. Hernandez, MD*** *Associate Professor of Urology* *Director, USF Urology Clinic South* *Urologic Malignancies, Robotic Surgery, BPH & Urolithiasis* *Chief of Urology, Tampa General Hospital* *2 Tampa General Circle, STC 6th floor* *Tampa, FL 33606, USA* *Fax: +1 813 250-2279* *E-mail: dhernan3@health.usf.edu*
